# Analysis of public search interest regarding government containment policy on COVID-19 new cases in Indonesia, Malaysia and Singapore

**DOI:** 10.34172/hpp.2021.46

**Published:** 2021-08-18

**Authors:** Muhammad Farid Rizqullah, Rizma Adlia Syakurah

**Affiliations:** ^1^Medical Faculty, Sriwijaya University, Indonesia; ^2^Public Health Faculty, Sriwijaya University, Indonesia

**Keywords:** COVID-19, Indonesia, Singapore, Malaysia, Policy

## Abstract

**Background:** As preventive measures to curb coronavirus disease 2019 (COVID-19)transmission, Indonesia, Malaysia and Singapore had imposed web-accessible regulations where the popularity of relative internet search volume can be obtained from Google Trends(GT). This research aimed to seek the relationship between public search interest and countries policies, furthermore to observe whether the GT data could be utilized as a tool to make a risk communication during this pandemic.

** Methods:** This retrospective study used GT to analyze the relative search volume (RSV) of keywords large-scale social restrictions (Pembatasan Sosial Berskala Besar – PSBB ), MovementControl Order (MCO) or Perintah Kawalan Pergerakan (PKP) and Circuit Breaker (CB) for Indonesia, Malaysia and Singapore respectively. Daily number of COVID-19 confirmed cases were collected and analyzed using Pearson correlation and time-lag with P<0.05. Every search interest peak and mobility trends changes were qualitatively analyzed.

**Results:** The results exhibited the relationship between the government containment policy, the peaks of analyzed RSV keywords and the mobility trends. The containment policy has significant relationships with COVID-19 daily cases (P<0.05).

** Conclusion:** These results indicated that the government could use GT RSV as a strategy of crisis and risk communication to intervene public behavior towards the pandemic.

## Introduction

The emergence of coronavirus disease 2019 (COVID-19) had brought a global catastrophe. The World Health Organization (WHO) announced COVID-19 as a pandemic on March 11, 2020.^1^ As of June 10, 2020, the disease that initially started in Wuhan, China had already spread and brought havoc on more than 200 countries with more than seven million confirmed cases worldwide.^2^ Due to its strong inter-connections with tourism and trade, countries in the South East Asia region were affected early.^3^ As the number of COVID-19 cases rapidly escalated, preventive measures must be promptly implemented. Several countries in South East Asia region namely Indonesia, Malaysia and Singapore had imposed regulations to prevent extensive spread of the disease, namely large-scale social restrictions (Pembatasan Sosial Berskala Besar – PSBB), Movement Control Order (MCO) or Perintah Kawalan Pergerakan (PKP) and Circuit Breaker (CB) respectively.^4^

Nowadays, information regarding COVID-19 and the policy taken by countries are accessible on the internet. One of the most popular search engines, Google, had developed a tool to measure the popularity of search terms on the internet, named Google Trends (GT).^5^ Information obtained from GT had already been used to study health phenomena in a variety of topic domains.^6^ The data from GT could also be used as a form of risk communication. Thus, it would be beneficial to deploy the information to the public regarding health events such as the COVID-19 pandemic. This research aimed to seek the relationship between public search interest and the policy taken by countries regarding COVID-19 in each country and to observe whether the GT data could be utilized as a tool to make a form of risk communication during this pandemic.

## Materials and Methods

### Study design

In this retrospective study, GT was used to analyze the volume of internet searches within the Indonesia, Malaysia, and Singapore regarding containment policy in each country such as “PSBB” for Indonesia, “MCO” and “PKP” for Malaysia, and “Circuit Breaker” for Singapore and COVID-19 daily cases, focusing on dates between December 31, 2019, and June 10, 2020. The volume of internet searches related to containment policy in each country compared to COVID-19 daily cases. GT number represents the relative search volume (RSV) to the highest point for the selected region and time. A value of 100 is the peak popularity of the term, whilst a value of 0 means the lowest popularity of the term.

### Data source

We used COVID-19 new cases daily reports from the official website of the WHO (https://covid19.who.int) that collected the COVID-19 cases data from all over the globe. The data used were time series data ranging from 31^st^ December 2019 to 10^th^June 2020.

### Data analysis

The data from GT were compared to the daily number of COVID-19 new cases in each country. Qualitative analysis was carried out in every peak of search interest and changes of mobility trend in each country. Quantitatively, the time-lag correlation was utilized to assess whether the rise of GT RSV data was correlated with the following increase of COVID-19 cases in each country.

## Results

**Figure 1:**
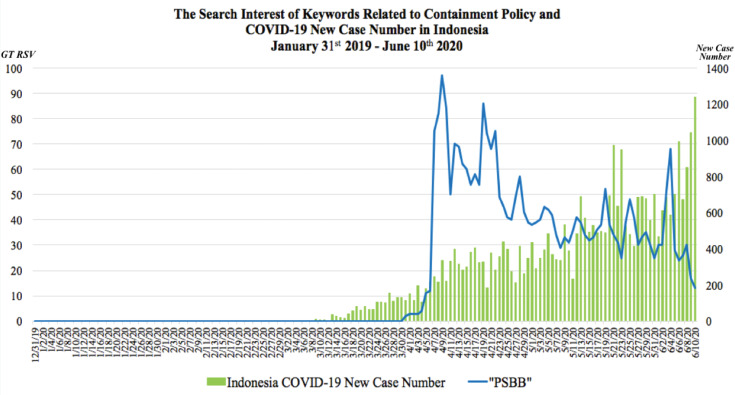
The comparison between the COVID-19 daily new cases and search interest of keywords related to containment policy in Indonesia on 31^st^ January 2019 until 10^th^June 2020

**Figure 2:**
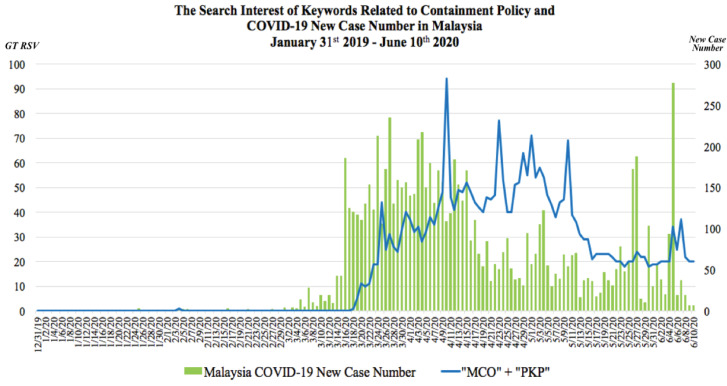
The comparison between the COVID-19 daily new cases and search interest of keywords related to containment policy in Malaysia on 31^st^ January 2019 until 10^th^ June 2020

**Figure 3:**
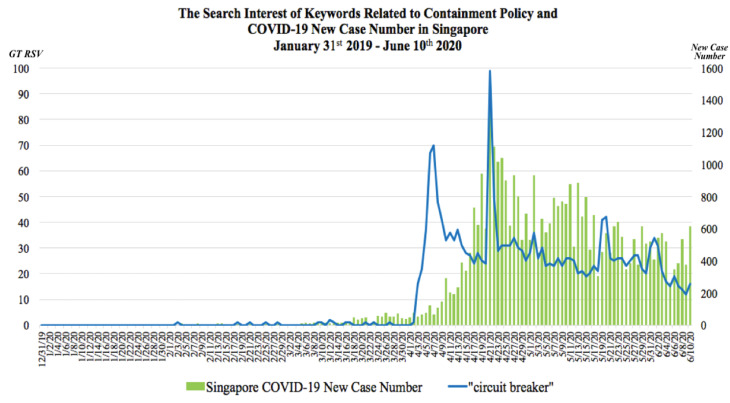
The comparison between the COVID-19 daily new cases and search interest of keywords related to containment policy in Singapore on 31^st^January 2019 until 10^th^June 2020

The GT RSV of keyword regarding containment policy and number of COVID-19 new cases in Indonesia, Malaysia and Singapore were presented in Figure 1, Figure 2 and Figure 3 respectively. In these graphs, the observable peaks represented surges of search interest on particular keywords regarding the containment policy in each country.

In Indonesia, seven peaks of search interest with keyword of “PSBB” were observed since the President of Indonesia signed the Government Regulation No. 21/2020 which regulated the large-scale social restrictions at the end of March. This regulation allowed regional governments that had been permitted by the Ministry of Health to limit movement of goods and people in their respective area.^7^ The search interest of “PSBB” started to increase sharply on April 7, 2020 as DKI Jakarta was approved to impose PSBB for 2 weeks since 10^th^April.^8^ The first three peaks of search interest were observed on 9^th^, 19^th^and 28^th^ April 2020 due to the implementation of PSBB in DKI Jakarta, followed by 2 provinces and 18 cities as well as regencies, and implementation of curfew in Surabaya city and its satellite regencies in East Java (Sidoarjo and Gresik) respectively.^9,10^ The capital city of Indonesia was a pioneer where this new regulation was implemented, seeking information on the internet helped reducing uncertainty about health issues as the announcement of the new regulations was made amid the COVID-19 pandemic.^9^ In addition, 28^th^ April 2020 was also the last day of PSBB in 5 cities and regencies (Bogor, Depok, and Bekasi) in West Java. Another peak was observed on 22^nd^ April 2020 which marked the day before the last day of phase 1 PSBB in DKI Jakarta. The other 3 peaks were observed on 19^th^ May, 25^th^ May and 4^th^ June 2020 which were due to the announcement of the regional government to extend the implementation of PSBB in the cities especially, DKI Jakarta, Surabaya and its regional government.^11,12^ The implementation of PSBB in DKI Jakarta was further extended to the end of June and was known as “PSBB Transisi” where the Ministry of Health had issued guidelines to reopen shops and offices to restore the economic balance.^12,13^

Meanwhile, the search interest in Malaysia regarding keyword “MCO” and “PKP’’ started to rise on 16^th^ March 2020 as the Prime Minister of Malaysia announced a nationwide Restriction of Movement Order from 18^th^ – 31^st^March 2020 (Figure 2). This measure was taken in response to a sharp rise in the number of COVID-19 cases. Some of the restrictions applied were the prohibitions of mass movements and gatherings across the country including religious, sports, social and cultural activities, restrictions on the entry of all tourists and foreign visitors into the country, closure of all public and private educational institutions as well as all government and private premises. People who were involved in essential services should undergo a health check and a 14-day quarantine for all Malaysian returning from abroad.^14^

Seven peaks of search interest were observed in Malaysia GT RSV data since the implementation of MCO. The first four peaks arose due to the repeated extension of MCO implementation announcements in Malaysia. In each cases, the Government of Malaysia decided to extend the MCO for another two weeks due to the spike of new positive cases in the COVID-19 trend in Malaysia, additional sanctions in the regulation, giving extra time for healthcare personnel in battling the COVID-19 outbreak along with reopening certain business sectors to sustain the country’s economy and ensure continuous access to basic needs and critical products.^15-19^ The following 3 peaks were observed on 1^st^ May, 10^th^ May and 7^th^ June 2020 regarding introduction of a plan named Conditional Movement Control Order (CMCO) which was a mitigation of the MCO regulations in order to reopen national economy in a controlled manner. Though CMCO regulation also experienced extension period, the Malaysian Government announced the end of CMCO and the country entered the Recovery Movement Control Order (RMCO) phase between 10^th^ June and 31^st^ August 2020.^20-22^

Search interest regarding the keyword “Circuit Breaker” started to rise in early April as the Prime Minister of Singapore announced a nationwide partial lockdown starting on 7^th^April–4^th^May 2020 after the increased number of cases over the preceding month as well as the risk of a huge cluster of infections (Figure 3).^23^Five peaks of search interest were observed as the Circuit Breaker regulation was implemented in Singapore. The first peak was observed on 7^th^ April 2020 which coincided with the first day of Singapore’s CB implementation. There was a restriction of movements, closure of school and implementation of home-based learning, non-essential workplaces closure; all religious activities were halted, all food establishments were only allowed to offer take-away, drive-thru and delivery of food. In addition, wearing mask in public was made compulsory.^24^ The next 3 peaks emerged on 21^st^ April, 2^nd^ May and 20^th^ May 2020 due to the Singaporean Government announcements of CB extensions along with stringent restrictions, CB measure mitigation and announcement to end CB respectively. During this period of time, the Government of Singapore had allowed reoperation of some non-essential businesses with limited workforces and reopened economic sectors in 3 phases.^25-28^ The last peak was observed on 1^st^ June 2020 which marked the last day of the CB period and entering the ‘safe reopening’, the first phase of post CB which started on 2^nd^ June. Singapore gradually re-opened economic activities that did not pose a high-risk transmission. Social, economic and entertainment activities with a higher risk remain closed.^29^

**Figure 4:**
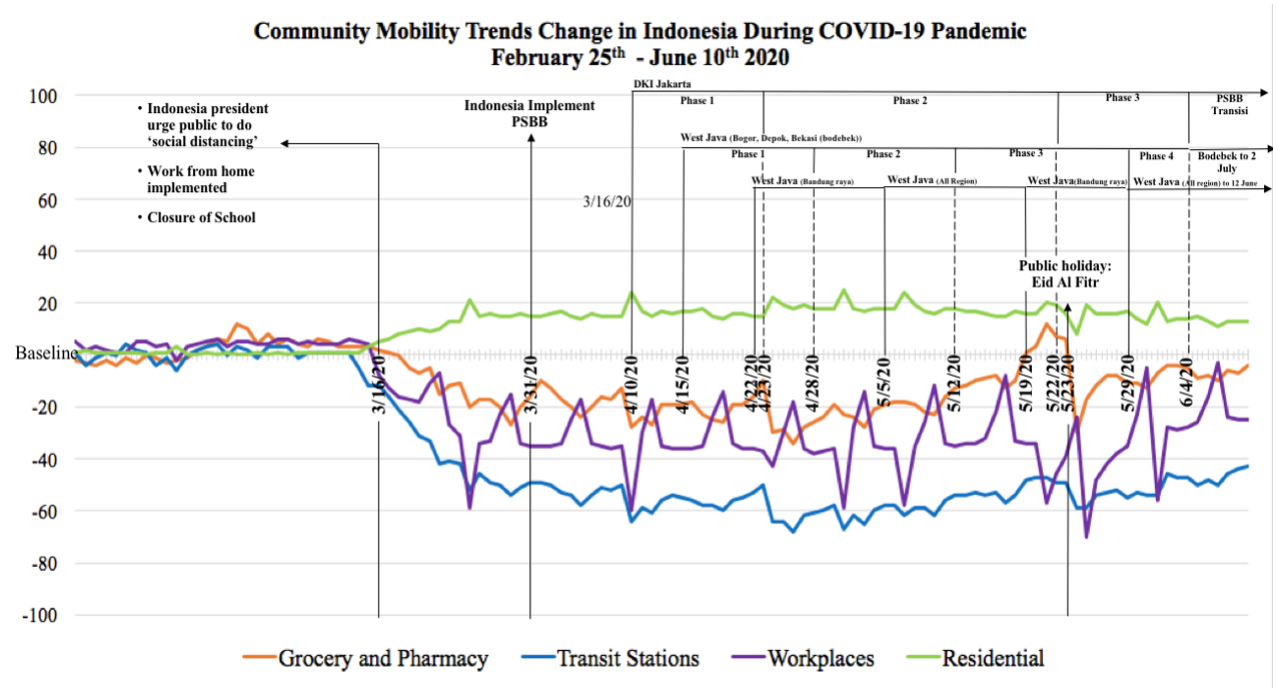
Changes of the mobility trends in Indonesia during COVID-19 pandemic compared to the baseline

**Figure 5:**
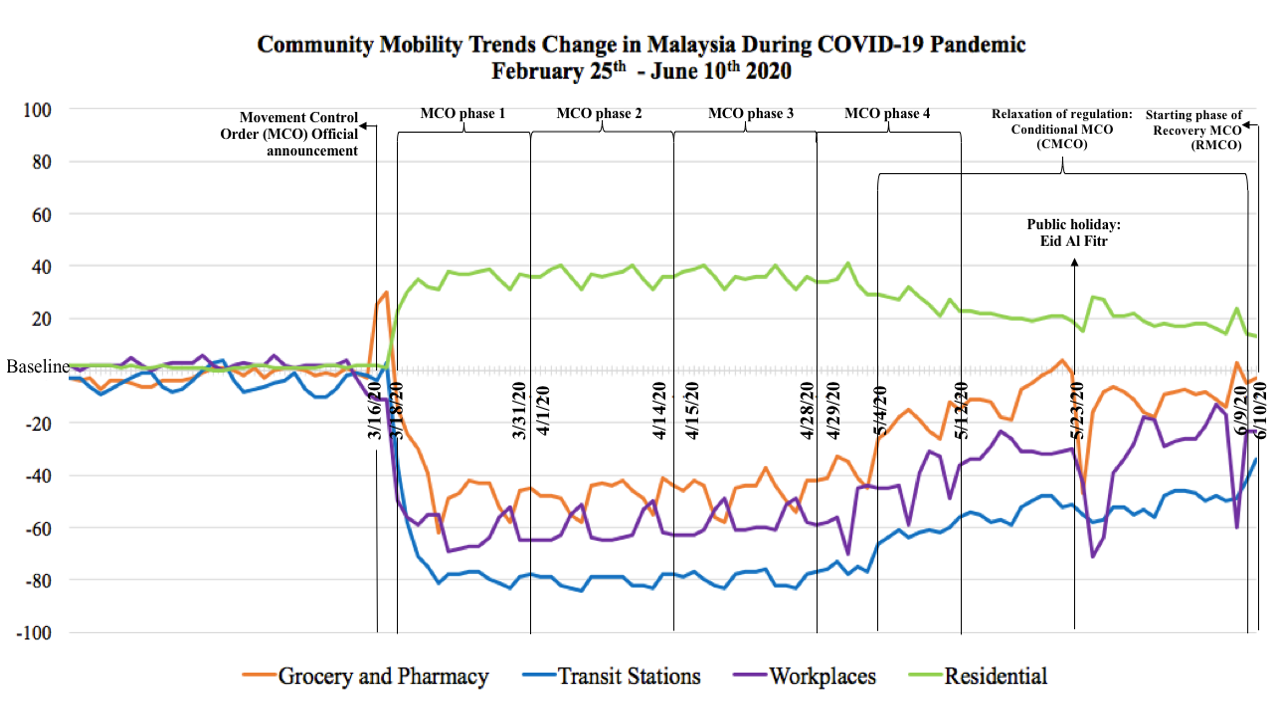
Changes of the mobility trends in Malaysia during COVID-19 pandemic compared to the baseline

**Figure 6:**
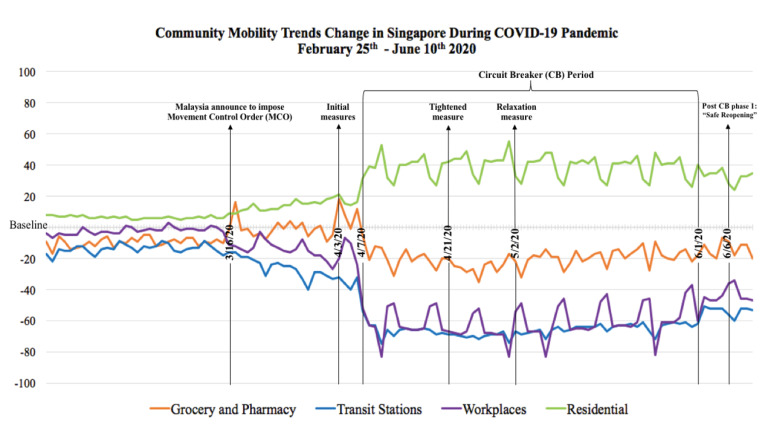
Changes of the mobility trends in Singapore during COVID-19 pandemic compared to the baseline

Furthermore, across Indonesia, Malaysia and Singapore, on 10^th^ June 2020, the reduction of people’s mobility in the visit to grocery and pharmacy ranged between 3% and 20%, transit station ranged 34% and 53%, workplaces ranged 23% and 37%, whereas the increase of being at residential places ranged between 33% and 35%, all with respect to the baseline determined by Google.^30^ The changes in community mobility trends in each country was shown in Figures 4–6.

The correlation between the RSV for containment policy and the number of daily COVID-19 confirmed cases in each country were shown in Table 1. Singapore had a significantly highest positive correlation (R = 0.741) compared to Indonesia (R = 0.606) and Malaysia (0.522). Time-lag wise, all keywords for containment policy in Indonesia and Singapore showed a significantly high positive correlation in all sets, while it showed a significantly moderate positive correlation in all sets for Malaysia. These results indicated that the increase in search interest regarding containment policy occurred three days prior, through the onset of a daily COVID-19 confirmed cases and stayed high for three days in each country.

**Table 1:** Result of time-lag Pearson correlations between keywords related to containment policy in Indonesia, Malaysia and Singapore GT RSV and COVID-19 daily cases in each country (31^st^ January 2019 until 10^th^ June 2020)

	**Days**						
Country (Search term)	Lag - 3	Lag - 2	Lag - 1	Lag 0	Lag + 1	Lag + 2	Lag + 3
Indonesia/"PSBB"	0.663*	0.677*	0.632*	0.606*	0.631*	0.637*	0.644*
Malaysia/"MCO + PKP"	0.458*	0.509*	0.515*	0.522*	0.564*	0.578*	0.594*
Singapore/“CB”	0.742*	0.719*	0.716*	0.741*	0.662*	0.673*	0.646*

*Significant with *P*≤ 0.05 Pearson correlation coefficient (R): 0-0.2, 0.21-0.40, 0.40-0.60, 0.61-0.80, 0.80-1

## Discussion

Based on the analysis, GT RSV data regarding keywords related to containment policy in Indonesia (PSBB), Malaysia (MCO and PKP) and Singapore (CB) could monitor the population reaction in each country towards the COVID-19 pandemic. As the number of COVID-19 cases increased, public attention towards the COVID-19 containment policy in Indonesia and Singapore had fluctuated from the start of the pandemic to June 2020 and declined afterwards, while it was stable in Malaysia. This showed that the public’s attention to the COVID-19 containment policy at the beginning of the pandemic was high, yet it was declining. Several factors, such as peer groups, mass media newsletters, government actions, and social media interactions could influence health-seeking behavior.^31^

Almost all public search interest rates in this research were always preceded by government announcements regarding COVID-19 containment policy, which suggested that government information disclosure helped focus public attention on the crisis. Policymakers may take advantage of this behavior to provide clear advices regarding the actions people can undertake to avoid risk.^32^ The government of Indonesia, Malaysia and Singapore provided plenty sources of reliable information, including social media equipped with hoax busters, websites, mobile applications, posters, videos, as well as hotlines and chatbots to the public.^33-36^

Monitoring community mobility trends change was essential as a country’s policy could affect the neighboring country’s mobility trends. Since the Malaysian government made an official announcement to implement MCO on 16^th^ March, there was an increase in visits to groceries and pharmacies both in Malaysia and Singapore. Similar trends happened in Malaysia and Indonesia before Eid Al Fitr, the most important Muslim celebration and usually proceeded by a culture to buy new clothes and gifts for the loved ones. The culture and policy were among components that affected the social and physical environment which determined the health of a community.^37^ Previous research on COVID-19 cases and effects on mobility trends at 127 countries proved that being at residential places had the highest impact on reducing COVID-19 deaths, followed by lesser visits to transit stations, retail & recreation areas, workplaces, parks as well as groceries and pharmacies store.^38^ Each country’s punishment for violations of containment policy also influenced the downturn of community mobility trends. People who violated the containment policy in Malaysia and Singapore were subjected to high penalty fines, prison sentences or sent to perform community services.^39-40^ However, violation of health protocol in Indonesia was only subjected to mild punishment such as written warning and a fine up to less than US$20.^41^

As of 30^th^ June 2020, Singapore case fatality rate (0.06%) was lower than Malaysia (1.04%) and Indonesia (5.09%).^42^ After the successful effort in combating severe acute respiratory syndrome (SARS) epidemics in 2002–2003, Singapore was hailed globally as a model of “flattening the curve” through its extensive testing, strict quarantine of infected cases and contact tracing of COVID-19.^40-43^ Singapore was also the first ASEAN country to establish a Multi-Ministry Task Force before the emergence of the first COVID-19 case in late January.^44^

Malaysia maintained a low number of daily confirmed cases before sudden outbreak due to mass religious gathering attended by approximately 16 000 people in late February 2020.^45^ The Malaysian government restricted a nation-wide movement, closed non-essential services and schools, and prohibited all kinds of gatherings until 30^th^ April 2020 in order to control number of COVID-19 cases. People who violated this regulation would receive a severe punishment. Malaysia’s nationwide response and collaboration could be a role model for other countries to flatten the curve of the COVID-19 pandemic.^33^

Indonesia had the greatest risk of suffering due to the inclining confirmed cases as a result of inadequate quality of health infrastructure. During the period of this study, Indonesia ranked the first for total number of confirmed cases and CFR in the ASEAN region. The Indonesian government established a task force to handle COVID-19 on 13^th^ March 2020 and began large-scale social restriction (PSBB) on 30^th^ March 2020 to accelerate the eradication of COVID-19.^45^ As of 7^th^ April, Indonesia’s testing capacity was on the fourth-worst testing rate, with only 36 in every million people being tested.^46^ The implementation of PSBB was not stringent enough as the punishments imposed were unable to make the public adhere with the containment policy. As of 30^th^ June, the confirmed case curve still showed no sign of flattening.

### Risk communication related to containment policy in Indonesia, Malaysia and Singapore GT RSV and COVID-19 daily cases

Risk communication is crucial to share vital information, maintain health, reduce losses, and giving respect to the public, also it needs monitoring as well as assessment of changes in society’s knowledge, habits, and behavior. Observations on movement of people and the increase cases of COVID-19 during the policy period showed the results of risk communication by the government.^47^ As government power upsurges during national public health emergencies, effective internal and external government communications involving the public, the media, organizations and other countries with similar health risks, are progressively important in appraising public audiences of impending threats and best practices to minimize menaces during the pandemic as well as stabilizing the society.^48,49^ Monitoring public responses to government policies about environment, natural resource management, energy, and contingency readiness can help to implement risk communication more effectively in order to stimulate behavior change and take protective action by influencing audience perceptions. The public can be involved in the risk management process to exchange information and common approaches to risk issues.^50^

Government communication is indispensable in the era of crisis. The government has greater responsibility to react and manage matters that threatened the public safety.^51^ The government is considered capable of improving institutional and community control through good risk management based on disaster mitigation efforts with the help of good risk communication. The reliable information sources from the government will generate public trust so that disaster mitigation will be easier to run.^52,53^ Based on the analysis, the government could utilize GT RSV to improve their efforts in analyzing and developing an integrated crisis and risk communication strategy that involves intervening with public behavior towards the pandemic.

Several limitations of this study that utilization of Google technology GT and Google Community Mobility Report (CMR)) may not be representing the whole of population of a country. Demographic groups that affected by COVID-19 such as the elderly and others who may be more prone to get the infection might not have internet access and may be underrepresented in such data. Second, the country-specific reasons were not considered; like sexual differences, age and economic in the make-up of Google users may occur between countries, thus affect country comparisons; such research requires further study and consideration of other socio-economic and psychological factors. Third, the method used here, examining associations between RSV, mobility, and case number, provides no insight into the causative factors driving such patterns. Fourth, the context of the RSV provide by GT is not clear. There are very many reasons for people to type in a term in Google Search. The Interests are by far not the only reason. There is no information about the person searching for the term, therefore this can not exclude. The data provided by Google CMR do not directly link to some COVID-19 containment measures. For example, ‘social distancing’ has widely promoted as a measure to reduce the transmission of COVID-19.^54^ Valenti et al used CMR data as an estimate of social distancing in the modelling of deaths in Brazil.^55^ However, CMR data provide no direct indication of adherence to such rules and indicate only general activity at specific location types.

The further studies use of GT as a tool to predict the health problem needs to be confirmed through official data for each country. Keyword searches through GT also need to be considered because of the influence of internet penetration in the region, digital literacy, health literacy and public perceptions of disease due to media exposure.

## Conclusions

Announcement and implementation of preventive measures taken by the government regarding containment policy to curb COVID-19 spread preceded every peak of public search interest and change in community mobility trends in Indonesia, Malaysia and Singapore. Based on the analysis, the government could utilize GT RSV to improve their efforts in analyzing and developing an integrated crisis and risk communication strategy that involves intervening with public behavior towards the pandemic.

## Acknowledgments

The authors would like to thank the supervisor and all staff of the Public Health Department, Faculty of Medicine, Sriwijaya University, Palembang, South Sumatera, Indonesia.

## Funding

Not applicable.

## Competing interests

Authors Muhammad Farid Rizqullah and Rizma Adlia Syakurah declare that they have no conflict of interest

## Ethical approval

No need for ethical approval as used of anonymous open data.

## Authors’ contributions

MFR and RAS responsible for the conception and design of the study. MFR performed literature search and statistical analysis. All authors performed data analysis, interpretation, and drafting. RAS did critical revision and final approval of the version to be published.
